# Conantokin-G Attenuates Detrimental Effects of NMDAR Hyperactivity in an Ischemic Rat Model of Stroke

**DOI:** 10.1371/journal.pone.0122840

**Published:** 2015-03-30

**Authors:** Rashna Balsara, Alexander Dang, Deborah L. Donahue, Tiffany Snow, Francis J. Castellino

**Affiliations:** 1 W.M. Keck Center for Transgene Research, University of Notre Dame, Notre Dame, IN, 46556, United States of America; 2 Department of Chemistry and Biochemistry, University of Notre Dame, Notre Dame, IN, 46556, United States of America; School of Pharmacy, Texas Tech University HSC, UNITED STATES

## Abstract

The neuroprotective activity of conantokin-G (con-G), a naturally occurring antagonist of N-methyl-D-aspartate receptors (NMDAR), was neurologically and histologically compared in the core and peri-infarct regions after ischemia/reperfusion brain injury in male Sprague-Dawley rats. The contralateral regions served as robust internal controls. Intrathecal injection of con-G, post-middle carotid artery occlusion (MCAO), caused a dramatic decrease in brain infarct size and swelling at 4 hr, compared to 26 hr, and significant recovery of neurological deficits was observed at 26 hr. Administration of con-G facilitated neuronal recovery in the peri-infarct regions as observed by decreased neurodegeneration and diminished calcium microdeposits at 4 hr and 26 hr. Intact Microtubule Associated Protein (MAP2) staining and neuronal cytoarchitecture was observed in the peri-infarct regions of con-G treated rats at both timepoints. Con-G restored localization of GluN1 and GluN2B subunits in the neuronal soma, but not that of GluN2A, which was perinuclear in the peri-infarct regions at 4 hr and 26 hr. This suggests that molecular targeting of the GluN2B subunit has potential for reducing detrimental consequences of ischemia. Overall, the data demonstrated that stroke-induced NMDAR excitoxicity is ameliorated by con-G-mediated repair of neurological and neuroarchitectural deficits, as well as by reconstituting neuronal localization of GluN1 and GluN2B subunits in the peri-infarct region of the stroked brain.

## Introduction

Cerebral ischemia, which is a consequence of loss of blood flow to the brain, triggers a cascade of molecular events, such as large inward currents in neurons, increased release of presynaptic glutamate that causes accumulation of extracellular glutamate, and subsequent hyperactivation of the postsynaptic glutamate/glycine-gated ion channels, specifically, the N-methyl-D-aspartate receptors (NMDAR). This glutamate excitoxicity leads to increased levels of neuronal intracellular Ca^2+^, mediating aberrant neuronal signaling by activation of caspases and calpains, and eventually resulting in neuronal dysfunction and cellular apoptosis [1, 2]. Thus, the NMDARs have been considered as drug targets in stroke. These receptors are voltage- and ligand-gated ion channels requiring glutamate and glycine as co-agonists for channel opening. The NMDAR allows influx of Na^+^ and Ca^2+^, which in-turn are involved in physiological and pathological events. The functional NMDAR is a heterotetramer composed of the ubiquitous GluN1 subunit, present as one or more of eight splice variants (a-h), and GluN2 subunits (A-D) that are expressed as independent gene products [3]. The type of GluN2 subunit present lends to the resultant ion channel its unique electrophysiological, pharmacological, and biochemical characteristics [4]. Although involved in physiological synaptic plasticity and memory formation, the GluN2B subunit also plays neuropathological roles in stroke, pain, Alzheimer’s disease, drug and alcohol dependency, and nociception [5], among others. Several NMDAR and GluN2B-specific antagonists that promote neuroprotection have been evaluated for their efficacy on animal models of stroke, but have met with limited clinical success [6]. Selfotel, a competitive NMDAR antagonist, showed neuroprotection in stroke models, but was ineffective in treating acute ischemic stroke in humans due to neurotoxic side effects [7]. Non-competitive inhibitors of the NMDAR, cerestat and remacemide, and NMDAR modulators, eliprodil and ifenprodil, were terminated from clinical trials due to lack of efficacy or psychotic side effects [8–11]. Aptiganel, another non-competitive NMDAR inhibitor, was neuroprotective in stroked rats, but exhibited untoward side effects on the central nervous system, and led to hypertension [12, 13].

A search to discover novel compounds that would modulate the activity of GluN2B-containing NMDAR channels led to traxiprodil, a derivative of ifenprodil. While this agent is well-tolerated and does not display many of the typical NMDAR antagonist-induced side effects, it failed to exert neuroprotection in clinical trials [14]. A distinctive family of peptides, the conantokins, present in the venom of *Conus* marine snails, inhibit ion flow through NMDARs [15]. These peptides contain γ-carboxyglutamate residues, which are essential for their antagonistic activities, and have been evaluated as potential neurotherapeutic agents. Conantokin-G (con-G), a complete gene product of *Conus geographus*, is a non-competitive inhibitor of NMDARs with selective inhibitory activity for the GluN2B subunit [16]. Con-G has been successfully tested as an anti-convlusant [17, 18] and as an anti-analgesic agent in animal models [19–21]. *In vitro* studies utilizing neuronal cultures have shown an anti-apoptotic mechanism of con-G [22], and also demonstrated con-G-mediated inhibition of extrasynaptic Ca^2+^ influx, which promoted ERK and CREB activation and thereby neuronal survival [23]. Con-G afforded neuroprotection in a rodent model of MCAO, displaying a reduction in the core infarct size accompanied by reduction of c-fos levels and increased Bcl-2 levels, thus attenuating delayed cell death [22, 24–26]. While pre-clinical models of NMDAR antagonists, including con-G, for stroke have been successful, there is a paucity of data correlating the effect of the antagonist on neuronal integrity and changes in cellular localization of NMDAR subunits in an ischemic setting. The current study focuses on assessing at the cellular level the effects of con-G on ischemia-mediated changes in neuronal integrity and on the cellular distribution of the NMDAR subunits at early (4 hr) and late (26 hr) postischemia timepoints.

## Materials and Methods

### Stroke Induction and Surgeries

All surgeries were performed on male Sprague-Dawley rats (220–270 g) using aseptic techniques. Animal experiments were performed according to the protocols approved by the IACUC of the University of Notre Dame (Protocol number 14–086). All rats were allowed food and water *ad libitum*. Middle carotid artery occlusion (MCAO) was induced in rats under isofluorane by inserting a 4–0 silicon coated monofilament occluder (Doccol, Sharon, MA) in the external carotid artery. The filament was kept in place for 2 hr, after which the rat was briefly re-anesthetized. The occluder was then retracted allowing reperfusion. The anesthetic inhibition by isoflurane of GluN1 or GluN2 subunits are reversible in less than 4 hr as determined by current recordings [27], well within the time when the animals were sacrificed.

### Conantokin-G Treatment

Con-G (GEγγL^5^QγNQγ^10^LIRγK^15^SN-NH_2_; γ = gamma-carboxyglutamate) was synthesized using solid phase peptide synthesis (Applied Biosystems, Model 433A) according to methods published earlier [28]. Intrathecal (i.t.) administration of 2 μM con-G in 0.9% saline was performed 30 min post-MCAO by implanting the catheter 3 days prior to the MCAO surgery. Although earlier studies [22, 25] utilized 0.001–2.0 nmol of con-G to determine its neuroprotective efficacy, their route of delivery was the more direct intracerebroventricular injection. Since our con-G administration method was i.t., a dose of 2 μM con-G was employed to establish the neuroprotective model. Furthermore, from our *in vitro* studies, we have observed that 2–5 μM conantokins could antagonize NMDA-evoked current and intracellular calcium influx in dissociated rat hippocampal or mouse cortical neurons [29]. The intrathecal catheter (Braintree Scientific, Braintree, MA) was inserted into the spinal column of the rat at the junction of the skull and 1^st^ vertebra while the rat was anesthetized with 100 μg ketamine/10 μg xylazine/gr weight and held in place on a stereotaxic frame. The catheter was stabilized with wound glue and a stainless steel pin plug at the open end of the catheter.

### Neurological Assessment

The assessment of neurologic score for each rat was based on the modification of a previously described procedure [30] as follows: (0) no signs, (1) failure to use left paw, (2) turning to the right, (3) circling, (4) inability to walk, and (5) death and were performed at 26 hr postischemia. A high neurological score indicated maximal neurological deficit. Significance was calculated by two-tailed unpaired *t* test.

### Measurement of Infarct and Swelling

Brains from rats that were sacrificed at 4 hr or 26 hr after induction of stroke (± con-G) were coronally sliced in seven 2 mm thick sections (approximately 4 mm anterior and 8 mm posterior from the bregma, labeled A-G), and stained with 2% 2,3,5-triphenyltetrazolium chloride (TTC) at 37°C for 30 min. Brain sections were taken 2 mm away from the rostral end after the olfactory bulb was removed and up to the corticocerebellar junction in a rat brain matrix (Kent Scientific, Torrington, CT). Images of TTC stained sections were employed to calculate infarct and swelling sizes by outlining sections and areas of infarcts that showed complete loss of TTC staining, as well as the noninjured contralateral hemisphere, using the Nikon Elements AR 3.2 software (Nikon). Total volume and infarct volume were calculated from the composite areas of sequential brain slices. The extent of cerebral swelling (S) was calculated as: S = (volume of ipsilateral hemisphere—volume of contralateral hemisphere)/(volume of contralateral hemisphere). The infarct size (IS), corrected for swelling, was calculated as: IS = (IS x contralateral hemisphere size)/(ipsilateral hemisphere size). TTC-stained brain sections were imaged on the Leica DFC420 gross microscope and images captured using the Leica FireCam 3.1 software. The images were processed to remove background utilizing Microsoft PowerPoint version 14.6.6.

### Histochemistry

TTC-stained brain sections fixed in 4% paraformaldehyde were embedded in paraffin, cut into 4 or 10 μm sections and stained with hematoxylin and eosin (H & E) for general cell morphology, von Kossa stain for calcium deposits, or Fluoro Jade B (Chemicon) for degenerated neurons. Prior to performing histochemical or immunohistochemistry stains, paraffin sections were warmed at 50°C for 30 min, then cooled and deparaffinized.

*Fluoro Jade B stains*: Deparaffinized sections (10 μm) were incubated in 0.06% potassium permanganate, rinsed in distilled water, and stained with 0.0001% Fluoro Jade B solution (Millipore). The slides were rinsed in distilled water, air-dried at room temperature overnight, cleared in xylene, and coverslipped with DPX (Sigma). Images were acquired using a 20X objective.

*Hematoxylin and eosin stains*: Deparaffinized sections (4 μm) were nuclear stained with hematoxylin 2, counterstained with eosin Y, cleared in xylene, and mounted with Cytoseal XYL medium (Richard-Allan Scientific, Kalamazoo, MI).

*Von Kossa stains*: Deparaffinized sections (10 μm) were incubated with 1% silver nitrate solution and exposed to 100W light for 2 hr. The sections were rinsed with distilled water, and the unreacted silver was removed by treatment with 5% sodium thiosulfate. The sections were counterstained with nuclear fast red, dehydrated with alcohol, and coverslipped with Cytoseal XYL medium.

*Periodic acid Schiff (PAS) staining for evaluating integrity of the cerebral vasculature*: Deparaffinized sections (4 μm) were oxidized with periodic acid for 5 min, rinsed in distilled water, and placed in McManus Schiffs reagent for 1 hr. The slides were rinsed in water, counterstained with Light green SF yellow, and mounted with Cytoseal. The vasculature basement membranes were stained magenta and the other tissue elements were stained green. PAS staining was employed to stain matrix-associated cerebral vasculature to identify compromised blood vessels [31, 32].

Brain sections stained with H&E, von Kossa stain, and Periodic Acid Schiffs (PAS) stains were digitally scanned at 40X magnification and imaged at 20X zoom using the Image Scope system (Aperio Technologies, Buffalo Grove, IL). For quantification of the von Kossa stains, grayish black Ca^2+^ microdeposits were averaged in the peri-infarct region from 3–5 fields on non-treated and con-G-treated rat brains at 4 and 26 hr postischemia. Images of Fluoro Jade B and all immunofluorescence stained sections were acquired on a Nikon TE 2000-S microscope using Nikon Elements AR3.2 software.

### Immunohistochemical Stains

The following primary antibodies were utilized for immunofluorescence staining of brain sections: monoclonal mouse anti-rat anti-MAP2 antibody (1.5 μg/ml) (Sigma, #M9942); monoclonal mouse anti-rat anti-GluN1 (5 μg/ml) (UC Davis/NIH NeuroMab facility, #75–272), monoclonal mouse anti-rat anti-GluN2A (5 μg/ml) (UC Davis/NIH NeuroMab facility, #75–288), and monoclonal mouse anti-rat anti-GluN2B (5 μg/ml) (UC Davis/NIH NeuroMab facility, #75–101). The secondary antibody was AlexaFluor647-donkey-anti-mouse IgG (1:500) (Invitrogen). Tissue sections were blocked with 5% normal donkey serum/0.3% H_2_O_2_ and then treated with 5% donkey serum/PBS. The antigens were labeled with the primary antibodies in 1% donkey serum/0.05% Triton X-100 overnight at 4°C. After washing in PBS, the sections were exposed to Image IT (Invitrogen) at 4°C, washed in PBS, and incubated in secondary antibody at room temperature. Hoescht 33258 was used to stain nuclei prior to mounting and coverslipping in ProLong anti-Fade medium. Fluorescence images were acquired on a Nikon TE 2000-S microscope using the Nikon Elements AR 3.2 software. Quantification of GluN1, GluN2A, and GluN2B of the peri-infarct regions was performed using the intensity profile module of Elements software, which is defined by a polyline of constant length (100 μm) over regions that showed distinct punctate staining. An intensity profile graph is generated along with the intensity values from which averages were calculated. Others have utilized similar procedures for determining puncta intensity [33, 34].

### Statistical Analysis

The number of rats for the different treatment groups were; n = 12 for non-treated stroked animals and n = 7 for con-G-treated stroked animals at 26 hr, and n = 4 for non-treated and con-G treated rats at 4 hr. All evaluations were performed blinded. Statistical data are presented as mean ± SEM. Statistical significance was determined by unpaired *t* test using Prism software 5 (Graph Pad software) and *p* < 0.05 was considered to be significant.

## Results

### Neurological Outcomes 26-Hour Post-con-G Treatment

**Fig 1:**
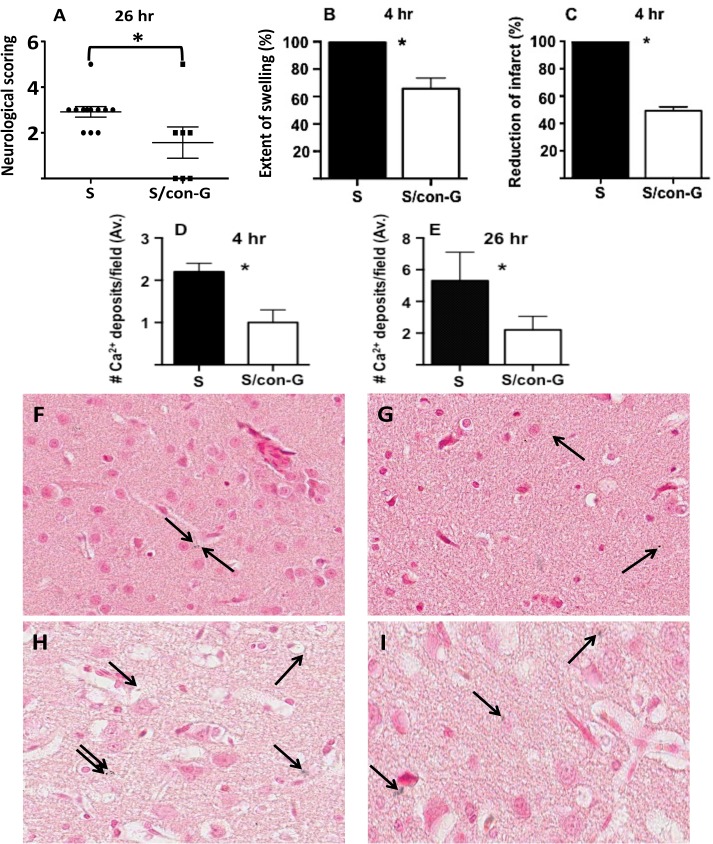
Treatment with con-G showed improved neurological scoring and decreased peri-infarct Ca^2+^ microdeposits. (A) Rats that underwent MCAO were either non-treated (S) or treated with 2 μM con-G (S/con-G) 30 min after stroke induction. Neurological assessments were performed 26 hr after ischemia induction Data are represented as mean ± SEM. *p = 0.037 between non-treated and con-G-treated stroked rats. (B) Intrathecal administration of con-G reduced swelling at 4 hr post-MCAO by ∼34%. (C) An approximate 50% reduction in infarct size was observed in the ischemic core in rats after 4 hr of con-G treatment. Changes in swelling and infarct sizes have been calculated as % of non-treated rats. (D) Coronal sections were processed with von Kossa stain for Ca^2+^ deposits. Ca^2+^ deposits were averaged from 3–5 peri-infarct fields of non-treated stroked rat brains (S) and con-G-treated stroked rat brains (S/con-G) at 4 hr and (E) 26 hr. Representative images of brain sections of the peri-infarct region stained for Ca^2+^ deposits by von Kossa staining from non-treated rats at 4 hr (F) and 26 hr (H) and con-G-treated brain rats at 4 hr (G) and 26 hr (I). Bars represent mean ± SEM. *p < 0.05 between non-treated and con-G treated group of rats. N = 12 for S and n = 7 for S/con-G at 26 hr, and n = 4 for both S and S/con-G at 4 hr.

The neurological outcomes of stroked rats were evaluated 26 hr after ischemia and i.t. delivery of 2 μM con-G, and compared to non-treated rats. The rodents were not neurologically graded at 4 hr after stroke induction and treatment, since their neuro-functional outcomes were minimal. However, at 26 hr, stroked rats not treated with con-G showed poor neurological outcome, with higher global neurological scores (2.91 ± 0.22), compared to con-G-treated rats (1.57 ± 0.63) (Fig 1A) and significant differences between non-treated and con-G-treated rats (*p* = 0.037). This indicated that treatment with con-G attenuated stroke-induced neurological deficits compared to non-treated rats.

### Brain Pathology: Infarct, Swelling, H&E Staining, and Neurodegeneration

The reduction in brain swelling by con-G was 34% compared to stroked rats that were not treated with con-G at the 4 hr timepoint (Fig 1B). Treatment with con-G 30 min after inducing stroke, significantly reduced the overall percentage of hemispheric infarct by 50% at 4 hr (Fig 1C). S1A and S1B Fig shows TTC-stained images of serial coronal brain sections of non-treated and con-G-treated rats, 4 hr post-MCAO and the actual infarct size (S1C Fig). Rats that were treated with con-G, and assessed at 26 hr, showed only a mild reduction in swelling (13.5%) compared to stroked rats that were not treated with con-G, and did not display an appreciable reduction in infarct size at 26 hr after con-G treatment (infarct reduction 4.3%). The inability of con-G to mitigate post-occlusion swelling at 24 hr has also been observed earlier [22], indicating that con-G was unable to sustain the decrement in hemispheric swelling due to edema at later timepoints.

Ischemia-induced damage was observed in the striatum and frontoparietal cortex and it was these areas of the brain that were histologically evaluated for con-G-meidated neuroprotection. Ischemia is also known to trigger cell death due to glutamate and calcium toxicity, which is enhanced late into the postischemic period in the cerebral cortex and hippocampus, promoting irreversible cell injury [35] and local inflammation [36]. Von Kossa stains of brain sections showed that Ca^2+^ microdeposits increased as a function of postischemic time. However, fewer Ca^2+^ deposits were observed in the peri-infarct regions of rat brains treated with con-G, compared to the peri-infarct regions of rat brains that were not treated with con-G at 4 hr and 26 hr (Fig 1D and 1E). (Fig 1F–1I) shows images of the peri-infarct regions of non-treated and con-G-treated rat brains at 4 and 26 hr postischemia from which average numbers of Ca^2+^ microdeposits were calculated.

**Fig 2:**
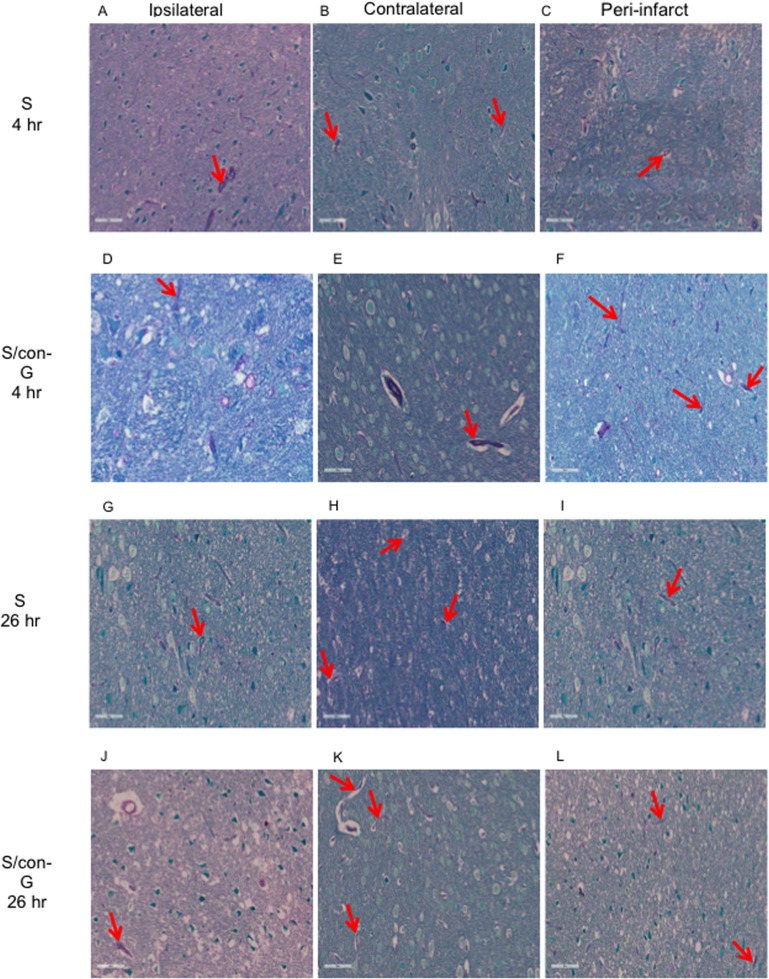
Effects of ischemia on cerebral vasculature. Stroke-mediated compromised vasculature was observed for non-treated (A-C) and for con-G treated rat brains (D-F) at 4 hr postischemia. Decreased vascular staining (red arrows) was observed in the ipsilateral regions of both non-treated (A) and con-G treated (D) stroked rat brains at 4 hr postischemia, and this observation is exacerbated in the ipsilateral region at 26 hr for both non-treated (G) and con-G-treated rat brains (J). The contralateral region shows intact vasculature at 4 hr (B, E) and at 26 hr (H, K) postischemia, while the peri-infarct regions showed vasculature that was between ipsilateral and contralateral at 4 hr in non-treated (C) and con-G-treated rat brains (F), and at 26 hr (I, L).

**Fig 3:**
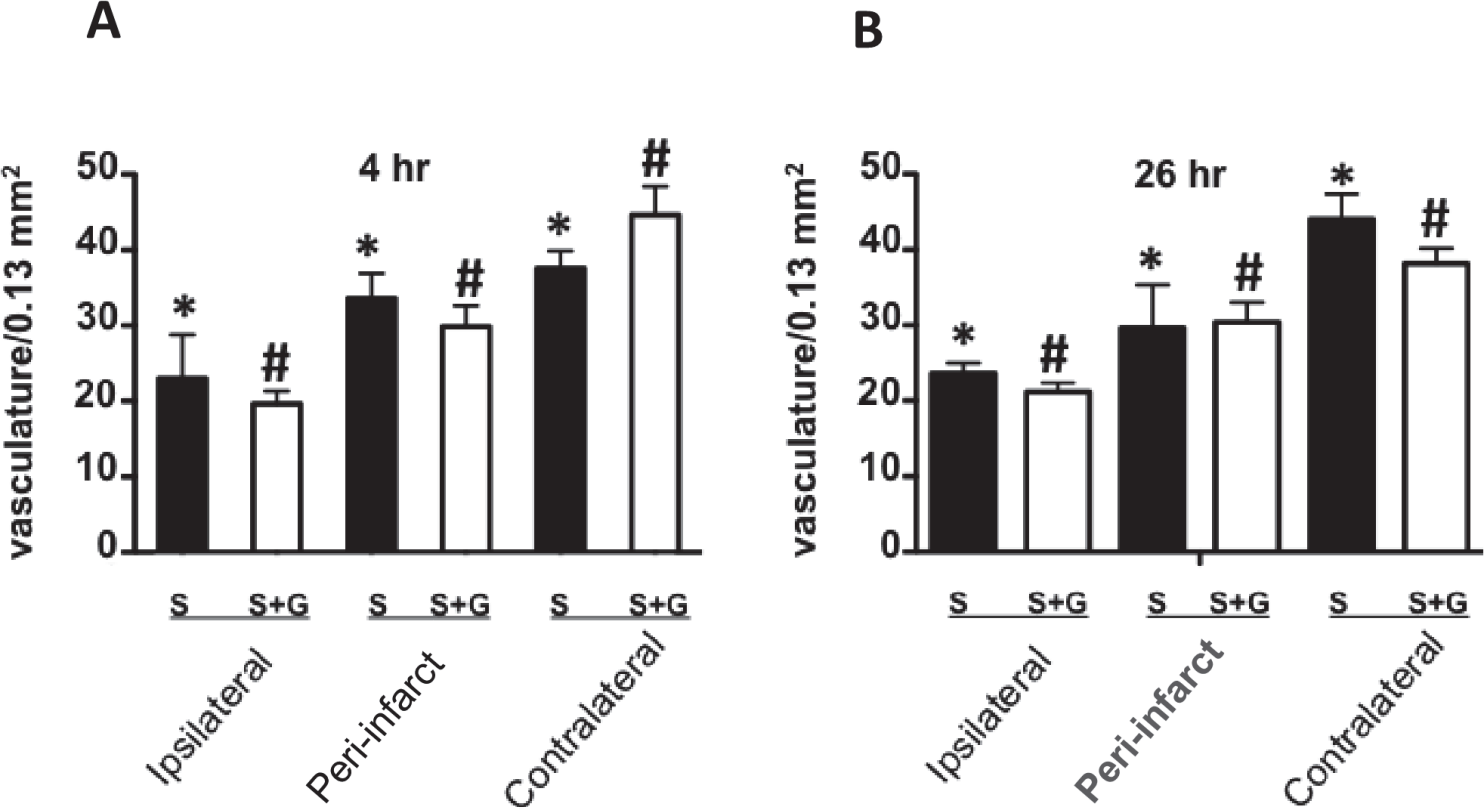
Effects of ischemia on numbers of cerebral vessels. Quantitation of the average vascular counts from 3–5 different fields of PAS-stained vasculature per 0.13 mm^2^ area at 4 hr (A) and 26 hr (B). * *p*<0.05 between stroked rats in the different regions of the brain, and # *p*<0.05 between con-G-treated rats in the different parts of the brain. Quantitatively, the peri-infarct regions showed average number of vasculature bodies that was between ipsilateral and contralateral regions in non-treated (black bars) and con-G-treated rat brains (white bars) at 4 hr and 26 hr. N = 12 for S and n = 7 for S/con-G at 26 hr, and n = 4 for both S and S/con-G at 4 hr.

**Fig 4:**
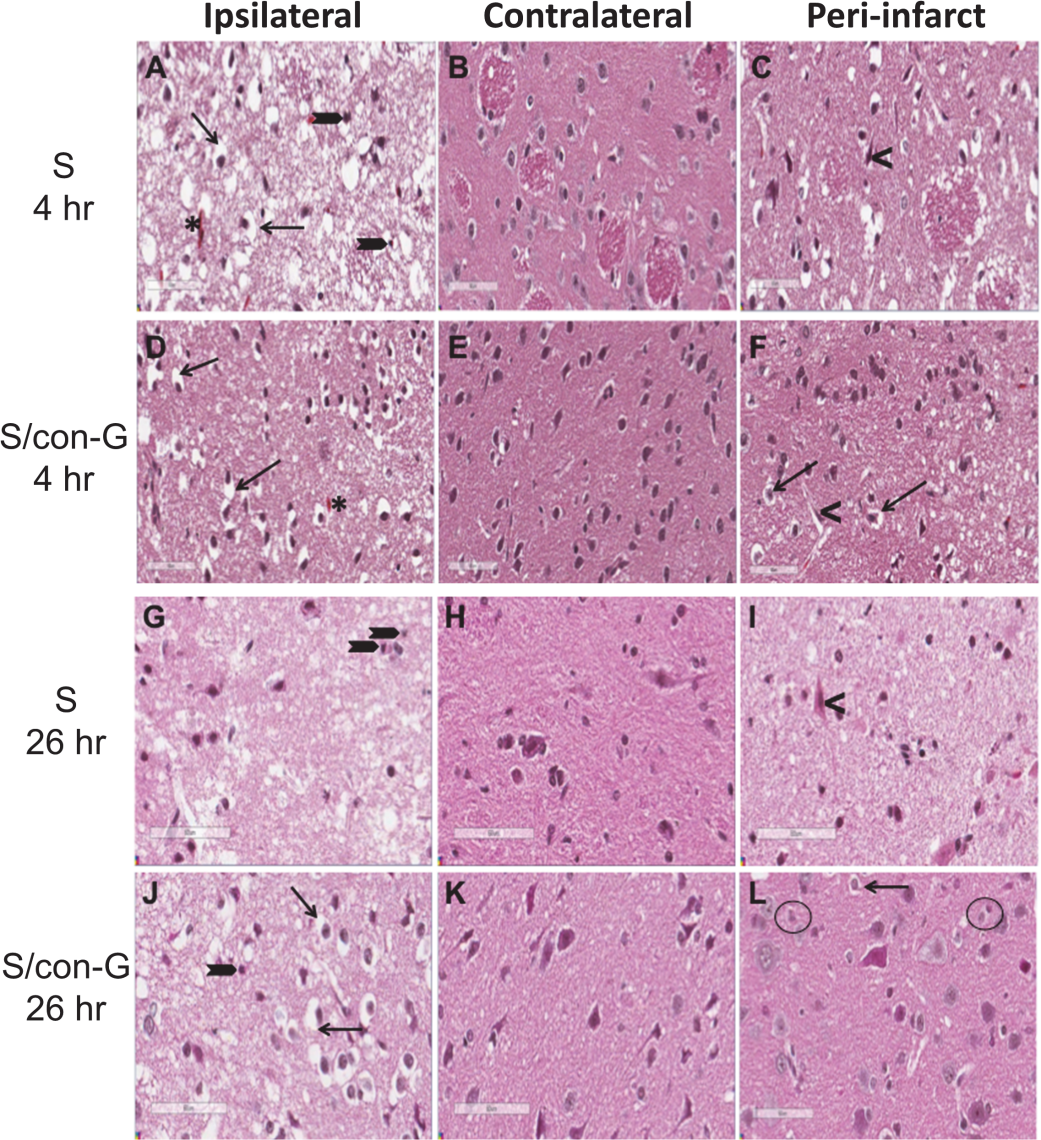
Con-G repairs MCAO-induced morphological changes. (A-L) Representative images of H & E stained coronal brain sections showing perturbed brain architecture of the ipsilateral side at 4 hr and 26 hr for non-con-G treated (A, 4 hr; G, 26 hr) and con-G treated rats (D, 4 hr; J, 26 hr), characterized by perineuronal vacuolation (black arrows) and shrunken neurons (block arrows), which is less dramatic at 4 hr (A) than at 26 hr (G). MCAO-induced pathology in the stroke area is similar in con-G treated brains at 4 hr (D) and 26 hr (J) compared to non-con-G treated brains. (B, E, H, K) show the normal brain architecture of the contralateral region of non-conG treated and con-G-treated rats at 4 hr and 26 hr after stroke induction. (C) The peri-infarct region at 4 hr and (I) 26 hr of con-G-treated and non-treated stroked rats with perineuronal vacuolation and degenerating neurons (<) observed. However, the peri-infarct region of con-G-treated rat brains shows repair of the brain cytoarchitecture at 4 hr (F). The perineuronal vacuolation, while present, is not as pronounced (arrows), with fewer degenerated neurons (<). (L) 26 hr con-G-treated rat brains showed enhanced cytorepair with unpronounced perineuronal vacuolation (black arrow) and diminished eosinophilic neurons (black circle). Total magnification, 200X. Bar scale, 60 μm. N = 12 for S and n = 7 for S/con-G at 26 hr, and n = 4 for both S and S/con-G at 4 hr.

Histochemical and immunohistochemical analyses were performed on the peri-infarct region, defined as the perifocal infarcted area surrounding the core white region (non-TTC stained) that is stained light pink [25], and has dysfunctional electrical activity, but remains viable [37]. These stains were compared to the contralateral region that served as an internal control for both non-treated and con-G-treated groups. The regions were also defined by PAS staining to determine cerebral blood vessel integrity to mainly demarcate the peri-infarct region from the core ipsilateral region. The ipsilateral regions showed significant diminished number of PAS stained vasculature compared to the contralateral regions in both, non-treated and con-G-treated rat brains (Fig 2). Whereas, the average density of vasculature in the peri-infarct regions was between of that observed in the ipsilateral and contralateral areas (Fig 3) for non-treated and con-G-treated rats at 4 hr and 26 hr. The ipsilateral regions of H&E stained sections at 4 and 26 hr post-MCAO showed perineuronal vacuolation that is indicative of swelling and degenerated dendrites, shrunken neurons with eosinophilic cytoplasm, and pyknotic nuclei for both, con-G non-treated and con-G-treated rats (Fig 4A, 4D, 4G and 4J). Some blood cells were also observed in the stroked area. No morphological changes were observed in the contralateral regions at the same time points (Fig 4B, 4E, 4H and 4K).

However, the peri-infarct region of the con-G treated stroked rat brains showed morphological repair with distinctly less neuropil vacuolation and shrunken neurons compared to the peri-infarct region of non-treated stroked rats at 4 hr (Fig 4C and 4F). At 26 hr, the peri-infarct region of non-treated rats displayed cytoarchitecture improvement (Fig 4I) compared to peri-infarct region of non-treated 4 hr (Fig 4C) brains due to a self-repair mechanism. This peri-infarct improvement is enhanced by con-G at 26 hr (Fig 4L),oHowH&E nnn with fewer perineuronal vacuolated and eosinophilic neurons with pyknotic nuclei.

**Fig 5:**
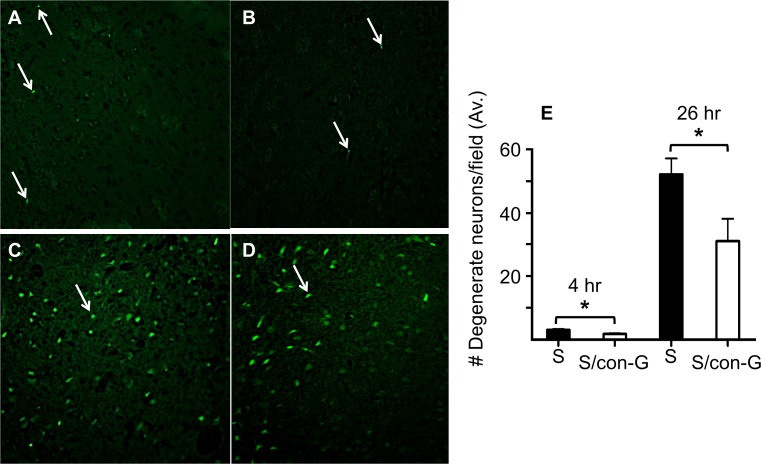
Con-G mitigates ischemic neurodegeneration and restores neuronal integrity. (A) Representative Fluoro Jade B stained sections of the peri-infarct regions of non-con-G treated rat brains (A) and con-G-treated rat brains (B) at 4 hr, and nontreated (C) and con-G-treated stroked rat brains (D) at 26 hr. Degenerated neurons are indicated by arrows. (E) Quantification of degenerated neurons averaged from 3–5 fields at 4 hr and 26 hr. *p<0.05 between non-treated and con-G-treated groups. N = 12 for S and n = 7 for S/con-G at 26 hr, and n = 4 for both S and S/con-G at 4 hr.

Brain sections of non-treated and con-G-treated stroked rats were stained with Fluoro Jade B to assess the extent of neurodegeneration. A time dependent increase in degenerated neurons was observed in the peri-infarct regions of both con-G non-treated and con-G treated rats. At 4 hr, weak Fluoro Jade B staining was observed in the peri-infarct region of both non-treated and con-G-treated rats (Fig 5A). At 26 hr, the peri-infarct area of non-treated rats showed intense Fluoro Jade B staining and an increased number of degenerated neurons compared to the 4 hr timepoint (Fig 5C and 5E). However, treatment with con-G diminished peri-infarct neurodegeneration at the 4 hr time point and this was sustained for 26 hr (Fig 5B, 5D and 5E). The amount of degeneration in the contralateral regions, which served as the internal negative control, of non-treated and con-G-treated brains was minimal (average of one degenerated neurons/field ± 0.55 at 4 hr, and two degenerated neurons/field ± 0.58 at 26 hr postischemia). Additionally, the average number of degenerated neurons in the core infarct of the ipsilateral regions of brains of non-treated and con-G-treated rats was also similar, but increased from 4 hr to 26 hr postischemia (average of 13.9 degenerated neurons/field ± 3.39 at 4 hr, and 116.3 degenerated neurons/field ± 7.24 at 26 hr postischemia, *p* < 0.001).

### MAP2 Staining for Neuronal Integrity

**Fig 6:**
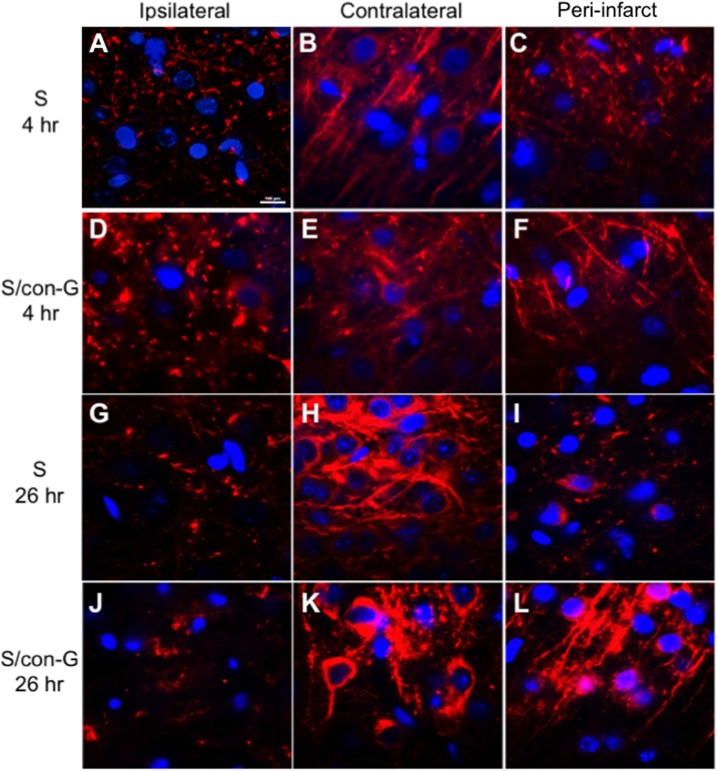
Anti-MAP2 staining in non-treated and con-G-treated stroked rat brains. At 4 hr postischemia, a lack of MAP2 immunoreactivity in the ipsilateral core of non-treated (A) and con-G-treated rats (D) was observed as compared to the contralateral regions (red staining), which exhibited robust MAP2 staining with distinct soma and dendritic structures in non-treated (B), and also in con-G-treated stroked rats (H). (C) The peri-infarct regions of non-treated stroked rats showed attenuated MAP2 immunoreactivity at 4 hr, but the peri-infarct regions of rats treated with 2 μM con-G demonstrated MAP2 staining in the soma and dendritic regions of the neurons at 4 hr (F). Similarly, at 26 hr postischemia, MAP2 staining was compromised in non-treated (G) and con-G-treated rat brains (J), compared to the contralateral side that showed intact MAP2 staining in the soma and dendrites (H, K) for both groups of animals. (I) The peri-infarct of non-treated rat brains did not show any recovery of MAP2 staining. (L) Con-G was able to sustain reinstitution of neuronal integrity at 26 hr as observed by MAP2 staining in the soma and dendrites. Bar scale, 100 μm for all images. N = 12 for S and n = 7 for S/con-G at 26 hr, and n = 4 for both S and S/con-G at 4 hr.

Microtubule-associated protein 2 (MAP2) is a cytoskeleton protein involved in maintaining neuronal integrity and modulating synaptic plasticity [38], and a loss of MAP2 is considered a hallmark for ischemic injury [39, 40]. At both 4 hr (Fig 6A) and 26 hr post-MCAO (Fig 6G), reduction in MAP2 staining in the ipsilateral infarct core region was apparent, and the cells differed in morphology from the neurons in the contralateral regions (Fig 6B and 6H) at the same timepoints. More densely stained soma and distinct dendritic structures for non-treated rats and con-G-treated groups of rats at 4 hr (Fig 6B and 6E) and 26 hr (Fig 6H and 6K) was observed in the contralateral regions. There is loss of MAP2 staining in the peri-infarct regions of non-treated stroked rats at 4 hr (Fig 6C), and is more obvious at 26 hr (Fig 6I). Rats injected with con-G demonstrated considerable neuronal integrity in the peri-infarct area at 4 hr, with distinct dendritic structures (Fig 6F). This repair of neuronal integrity was sustained until 26 hr in the peri-infarct region (Fig 6L). Thus, administration of con-G is able to repair MCAO-mediated aberrant neuronal morphology, and this was observed in all the rats treated with con-G at both timepoints.

### Changes in Localization Patterns of GluN Subunits

**Fig 7:**
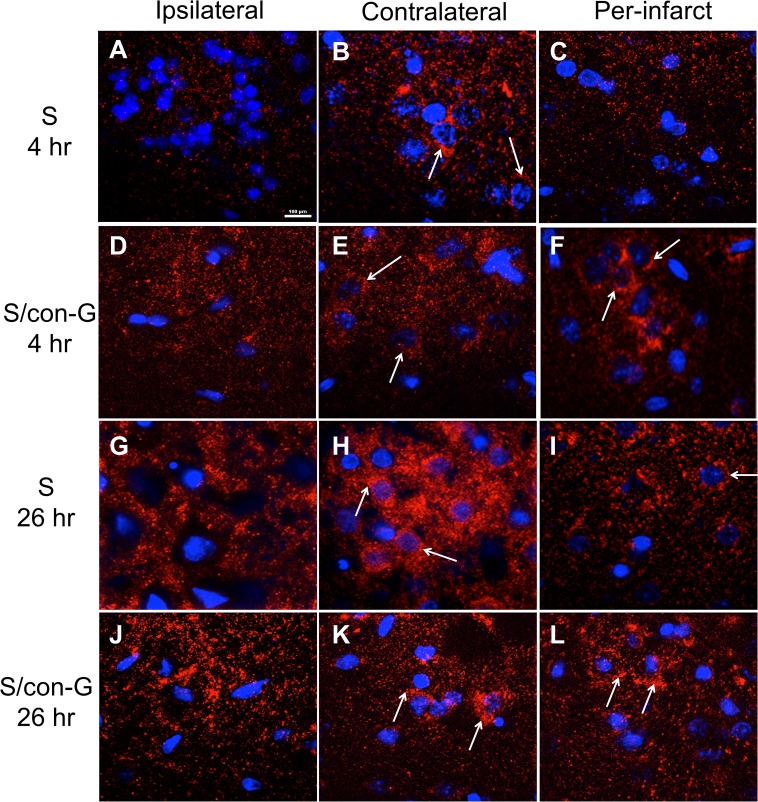
GluN1 localization pattern in non-treated and con-G-treated stroked rats at 4 hr and 26 hr. Random punctate staining of GluN1 (red) observed in the ipsilateral regions of stroked rat brain sections at 4 hr (A, D). In the contralateral regions of non-treated (B, 4 hr; H, 26 hr) and-con-G treated stroked rat brains (E, 4 hr; K, 26 hr), distinct GluN1 staining around the soma (blue) is observed (arrows). At 4 hr, the peri-infarct regions of non-treated brains showed similar random punctate staining of GluN1 (C) as the ipsilateral regions; however, the conG-treated stroked rats showed GluN1 staining around the soma (arrows) (F). At 26 hr, the ipsilateral regions of non-treated and con-G-treated-stroked rat brains showed loss of GluN1 staining around the soma (G, J), compared to the contralateral regions (H, K). At 26 hr, the peri-infarct region of non-treated stroked rat brains showed punctate GluN1 staining in the soma (arrow) (I), but the con-G-treated rat brains showed concentrated clusters of somal GluN1 (arrows) (L). Magnification, 100X. Bar scale, 100 μm for all images.

GluN1, GluN2A, and GluN2B are the prominent subunits of the functional heterotetrameric channel in the mature forebrain [41]. Therefore, we examined ischemia-mediated changes in the localization of these subunits, and the degree to which con-G is able to reverse any observed changes. Localization of the GluN1 subunit was dispersed after 4 hr of MCAO in the ipsilateral and peri-infarct regions of non-treated rats (Fig 7A and 7C), compared to the contralateral areas (Fig 7B), where some GluN1 clustering around the soma is observed. In the presence of con-G in stroked rats at the 4 hr timepoint, the ipsilateral region still shows punctated GluN1 expression (Fig 7D), compared to the contralateral region (Fig 7E). Additionally, somal clustering of GluN1 recurs in the peri-infarct area (Fig 7F), suggesting a protective effect.

At 26 hr post-MCAO, the ipsilateral regions of both non-treated and con-G-treated rats were similar, showing loss of GluN1 subunit immunostaining (Fig 7G and 7J). The neurons in the contralateral regions showed concentrated clusters of GluN1 subunit in the soma (Fig 7H and 7K). The peri-infarct regions of the non-treated stroked rat brains displayed punctate staining of the GluN1 subunit, indicative of the occurrence of a natural recovery process (Fig 7I), but the GluN1 subunit expression was more pronounced in the peri-infarct field of con-G-treated rat brains, and resembled the pattern observed for the contralateral region (Fig 7K and 7L). S2A and S2B Fig demonstrates the method utilized to determine puncta intensity, showing as an example the image of GluN2A immunofluorescence (S2A Fig) and its corresponding intensity profile (S2B Fig). Since, our focus is on the redeemable peri-infarct region, it was observed that GluN1 puncta intensity increased in con-G administered rat brains at both 4 hr (S2C Fig) and 26 hr (S2D Fig).

**Fig 8:**
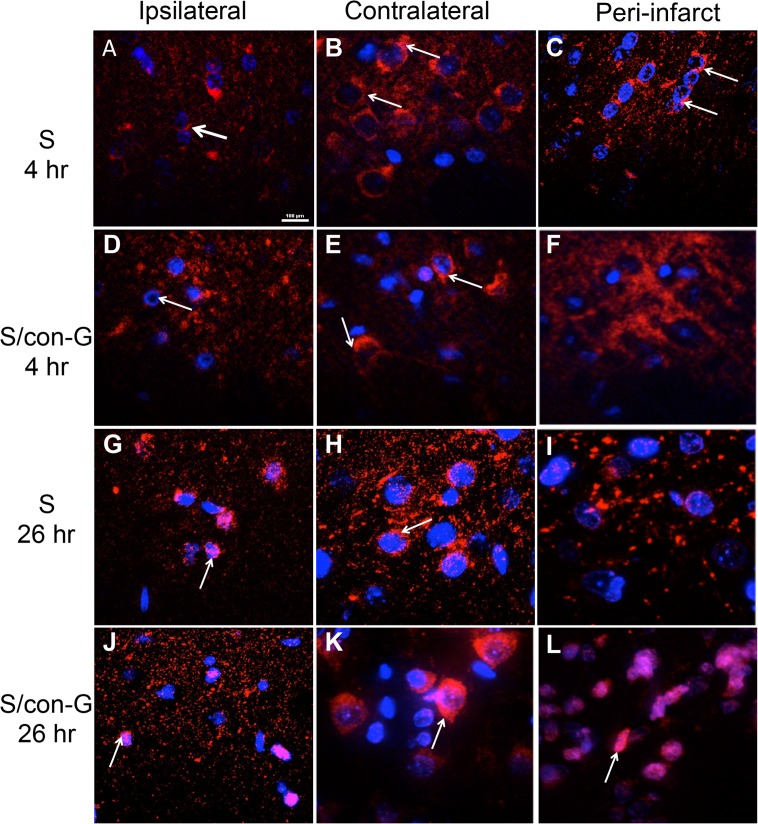
Effect of con-G on GluN2A localization patterns. Distinct GluN2A localization around the soma was disrupted by MCAO in the ipsilateral region (A, D) compared to the contralateral region (arrows) (B, E). Clusters of GluN2A were observed in the peri-infarct region of non-treated stroked rat brains (arrows) (C), but this was not observed in con-G-treated brains at 4 hr (F). At 26 hr post-MCAO, perinuclear staining of GluN2A was observed (arrows) (G, J) in the ipsilateral region compared to GluN2A staining observed in the soma of the contralateral region (arrows) (H, K). The peri-infarct of non-treated stroked rat brains showed no specific GluN2A staining (I), but the con-G-treated rat brains showed increased perinuclear presence of GluN2A (arrow) (L). Magnification, 100X. Bar scale, 100 μm for all images.

After 4 hr or 26 hr post-MCAO induction, loss of localization of the GluN2A subunit around the neuronal soma was observed in the ischemic core region (Fig 8A and 8G), compared to the contralateral areas (Fig 8B and 8H). At 26 hr post-MCAO, perinuclear staining of GluN2A, with or without con-G treatment, was observed (Fig 8G and 8J), compared to the neurons in the contralateral region. Here, the GluN2A subunit is visualized in the soma, as well as in the proximal dendrites (Fig 8H and 8K). At 4 hr, in the peri-infarct region of non-treated stroked rat brains, clusters of GluN2A localization were observed polar to the condensed nucleus (Fig 8C), similar to the contralateral regions (Fig 8B and 8E). However in the peri-infarct region of con-G-treated brains, no such polar clusters of GluN2A were observed (Fig 8F). This indicated that con-G did not have an effect on GluN2A localization pattern at the 4 hr timepoint. At 26 hr post-MCAO, the peri-infarct area of non-treated rat brains showed little staining for GluN2A subunits (Fig 8I), whereas the con-G-treated peri-infarct area of stroked rat brains showed a distinct perinuclear GluN2A expression pattern (Fig 8L). These observations suggested that con-G did not restore the GluN2A localization pattern at the 4 hr timepoint, but directed perinuclear trafficking of the GluN2A subunit at the later time of 26 hr. Quantification of the GluN2A puncta in the peri-infarct regions showed that there was a slight, but not significant, increase in GluN2A intensity at 4 hr in con-G-treated animals, compared to non-treated stroked rat brains (S2E Fig). However, there was significant increase in puncta fluorescence intensity of GluN2A at 26 hr in the peri-infarct region of con-G-treated rat brains that was perinuclear in localization (S2F Fig).

**Fig 9:**
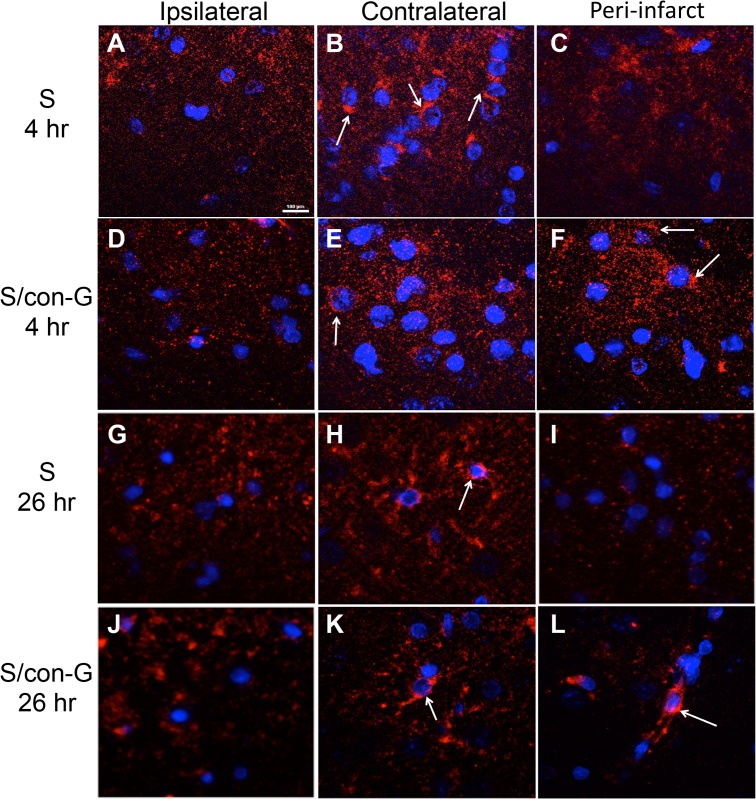
Con-G restores GluN2B localization at 26 hr. Loss of GluN2B was observed in the ipsilateral regions at 4 hr (A, D) and 26 hr (G, J) compared to the contralateral regions, where polar (B; arrows) or somal (blue) localization (E, H, K; arrows) of GluN2B was observed. The peri-infarct region of untreated rat brains lacked specific GluN2B staining at 4 hr (C) and 26 hr (I), but a distinct GluN2B presence (red) was observed in the peri-infarct area of con-G-treated rats at 4 hr (F) and at 26 hr (L). Magnification, 100X. Bar scale, 100 μm for all images.

The GluN2B subunit 4 hr after MCAO showed a random clustering pattern in the ischemic core (Fig 9A and 9D), whereas the contralateral regions showed either polar localization of GluN2B in the soma (Fig 9B), or concentrated clustering around the soma (Fig 9E, 9H and 9K), depending on the particular imaged contralateral region. The peri-infarct region of untreated rat brains exhibited a similar localization pattern as the ipsilateral region at 4 hr (Fig 9C) and 26 hr (Fig 9I), but the con-G treated rat brains showed selective clustering around the soma (Fig 9F) at 4 hr, which became more distinct at 26 hr (Fig 9L). Puncta fluorescence intensity of GluN2B was decreased in the peri-infarct region at 26 hr of non-treated stroked rat brains compared to 4 hr, but that significantly increased at 26 hr in con-G-treated brains compared to non-treated rat brains (S2 G and S2 H Fig). We are confident that the GluN subunit staining patterns observed are neuronal from their distinct morphology, and rat astrocytes do not transcribe GluN1 subunits and hence cannot form functional channels [42]. Furthermore, oligodendrocytic NMDARs are mostly comprised of NR1, NR2C, and NR3A subunits and these cells reside in the cerebellum and corpus callosum, which are not regions included in our analysis [43].

## Discussion

In this study, the effects of con-G on MCAO-induced neurological damage at the cellular and histological levels were evaluated. More specifically, a focus was placed on the therapeutic effects of con-G on injured neuron morphology and localization patterns of the GluN1, GluN2A, and GluN2B subunits in the peri-infarct regions, since this area is considered a potential target for neuroprotective drugs due to low levels of metabolic activity preserved [44, 45]. These observations were compared to those seen in the ipsilateral area and regions contralateral to the infarct area. The study was performed at timepoints of 4 hr and 26 hr post-MCAO, since there is a linear progression from initial injury to final infarct formation during this period. These timepoints also allowed the determination of the extent to which con-G retains its therapeutic effects, especially on the GluN subunit localization patterns that could possibly determine the ability of the cell to return to normal neurological functioning. Significant reductions in MCAO-induced swelling and infarct volumes were observed in con-G-treated rats at 4 hr, but this reduction was not sustained at the 26 hr timepoint. It has been demonstrated [22] that a single con-G application (0.5 nmol at 30 min post-MCAO) decreased the total and core infarct volumes, but not edema, at 72 hr. We observed con-G dependent improvement in neurological scoring at 26 hr, which is consistent with published data [22]. At both timepoints, of 4 and 26 hr, con-G administration effectively repaired MCAO-induced damage to the brain cytoarchitecture, as well as decreased the number of degenerated neurons and Ca^2+^ microdeposits in the peri-infarct region. This supports the role of con-G as a neuroprotective agent. Earlier studies with con-G demonstrated that this agent mitigated MCAO-induced DNA damage by increasing Bcl-2 levels and decreasing c-fos levels [25]. However, the effect of con-G on the various NMDAR subunits that are pathologically activated during ischemia was not evaluated in previous work.

Changes in NMDAR subunit expression levels in the brain during ischemia are dependent on the type of injury/reperfusion model, the time points studied, and the region of the brain evaluated. A significant reduction in GluN2A mRNA levels was observed in the hippocampal area and dentate gyrus of stroked rats [46], and mRNA levels of GluN1, GluN2A, and GluN2B decreased more dramatically in the caudate putamen, and, to a lesser extent, in the cerebral cortex. A previous study [47] has reported decreased levels of GluN2B subunit in the Triton-insoluble fraction/postsynaptic compartment of the ipsilateral and peri-infarct tissues at 4 hr and 48 hr postischemia. While the GluN1 levels were not affected, GluN2A levels were diminished in the ischemic core at 48 hr. Our studies showed that the distinct localization of the GluN1, GluN2A, and GluN2B subunits that was seen in the contralateral regions was not observed in the infarct of the ipsilateral regions at 4 hr and 26 hr after stroke. Administration of con-G restored the localization of GluN1 and GluN2B subunits, but not the GluN2A subunit, at 4 hr and 26 hr. This could explain the molecular corrections in neurological deficits at 26 hr post-MCAO, although gross reduction in infarct and swelling was not observed at this timepoint. Decreased GluN2B subunits resulted in impaired NMDAR-directed LTP and neurological deficits, such as spatial learning [48], whereas, overexpression of the GluN2B subunit improved synaptic plasticity and memory [49]. Our histological analyses show that con-G reconstituted the postischemic decrease of GluN2B subunits in the peri-infarct at 26 hr, which coincides with corrective neurological function. It has been demonstrated that ifenprodil, a GluN2B-specific antagonist, blocked postischemic long-term potentiation (LTP), without affecting activity-dependent LTP. Further, ifenprodil treatment allowed recovery of postsynaptic GluN2B levels in the infarct core, but did not reinstitute GluN2A levels [47]. By selectively blocking the GluN2B subunit with ifenprodil, it was observed that ischemic neuronal death was attenuated, whereas selective blocking of the GluN2A subunit with NVP-AAM077, exacerbated ischemic neuronal death with subsequent downregulation of CREB-directed *cpg15* and *bdnf* mRNA levels [50]. An alternate approach to the traditional pharmacological antagonism of NMDAR has been vaccination to generate autoimmune responses targeting the GluN1 subunit. It has been shown that GluN1 vaccinations were able to suppress kainate-induced seizures, as well as be neuroprotective in MCAO-induced stroke, decreasing total infarct volume in the cortex and striatum [51]. One possible mechanism of this immune-derived neuroprotection against MCAO damage is the inability of GluN1 to interact with tPA reducing excitotoxic insults of ischemia [52, 53]. Thus, antagonism of the GluN1 subunit is beneficial against neurodegenerative diseases by allowing the ion channel to be blocked by the Mg^2+^ ion and preventing excess Ca^2+^ influx (http://www.jn-vaccines.org/Stroke_Vaccine-JNIMC.pdf).

Proper anchoring and distribution of the NMDARs is critical for transmission of synaptic signals. In our model, at 26 hr after MCAO, GluN2A showed distinct perinuclear staining in the infarct core region and this was enhanced in the peri-infarct by treatment of con-G. This indicated that con-G failed to target corrective mobilization of GluN2A to the soma. However, con-G effectively reversed MCAO-induced loss of neuronal integrity as observed by MAP2 staining in the peri-infarct region. The ability of con-G to restore localization of GluN1 and GluN2B subunits, combined with decreased load of Ca^2+^ microdeposits, indicates that normal NMDAR/Ca^2+^ signaling events can occur leading to neuronal survival in the peri-infarct region. Our current data is in agreement with previously published work that showed that disruption of neuronal integrity due to GluN2B-dependent extrasynaptic activation was addressed most efficiently by con-G [23]. Since ischemia-induced excess glutamate activates the extrasynaptic NMDARs, which is composed mostly of the GluN2B subunit, con-G treatment restored neuronal integrity. Other neuropathologies such as, Alzheimer’s disease, Parkinson’s disease, and mood disorders that involve dysfunction of the NMDARs have reported altered function and subcellular localization of the receptor that is attributed to prolonged activation of the NMDAR [54]. In a rat model of Parkinson’s disease, it was observed that treatment with L-DOPA altered the synaptic localization of GluN2B and that dyskinetic animals had significantly higher amounts of GluN2A in the postsynaptic compartment [55]. Thus, pharmacological manipulation of the NMDAR, specifically, the GluN2B subunit that influences cellular localization is important for neuronal responsiveness and survival. NMDAR activity is a consequence of its subcellular location [56, 57], which makes them respond differentially to conantokins when neuronal NMDARs are pathologically stimulated [23]. Future plans involve determination of ischemia-driven changes in subcellular location of GluN subunits at the synaptic level and correlating that to aberrant electrophysiological activity of the ion channel in the presence or absence of con-G.

## Conclusion

In conclusion, we have demonstrated that the GluN2B-specific conantokin, con-G, corrected MCAO-induced neurological deficits, as well as structural changes to the neurons and neuronal cytoarchitecture due to ischemia in the peri-infarct region of the stroked brain. Ischemia-mediated changes in the localization patterns of GluN1 and GluN2B subunits were restored by treatment with con-G in the peri-infarct regions, indicating that the neuroprotective effect of con-G was achieved *via* NMDARs. By antagonizing GluN2B in the MCAO model, decreased ischemia-mediated Ca^2+^ load and degenerated neurons were also observed.

## Supporting Information

S1 Fig Administration of con-G decreases infarct size after 4 hr of MCAO. (A) Serial coronal sections of the same brain stained with TTC for non-treated rats and (B) rats treated with 2 μM con-G sacrificed 4 hr post-MCAO. The infarct area is observed in white in the right hemisphere (black arrows). (C) Actual infarct size for non-treated and 2 μM con-G treated rat brains. N = 4 for both S and S/con-G at 4 hr. *p<0.05 between non-treated and con-G-treated groups.
(TIF)

S2 Fig Quantification of GluN1, GluN2A, and GluN2B subunits in the peri-infarct regions of non-treated and con-G-treated stroked rat brains. (A) Representative image of GluN2A staining in the peri-infarct region of non-treated rat brains to show the 100 μm polyline for determining fluorescence intensity profile. (B) In the intensity profile, red indicates GluN2A subunit fluorescence intensity over a distance of 100 μm and the blue indicates intensity of the nuclear stain DAPI. Quantification of the GluN1 punctae at 4 hr (C) and 26 hr (D), of GluN2A staining at 4 hr (E) and 26 hr (F), and of GluN2B staining at 4 hr (G) and 26 hr (H). *p<0.05 between non-treated (S) and con-G (S/con-G) treated rat brains. N = 2 for S and n = 7 for S/con-G at 26 hr, and n = 4 for both S and S/con-G at 4 hr.
(TIF)
