# Supplementary figures and images for: Conantokin-G Attenuates Detrimental Effects of NMDAR Hyperactivity in an Ischemic Rat Model of Stroke
Source: PLoS One. 2015 Mar 30;10(3):e0122840. doi: 10.1371/journal.pone.0122840 (PMC4379059; doi:10.1371/journal.pone.0122840)

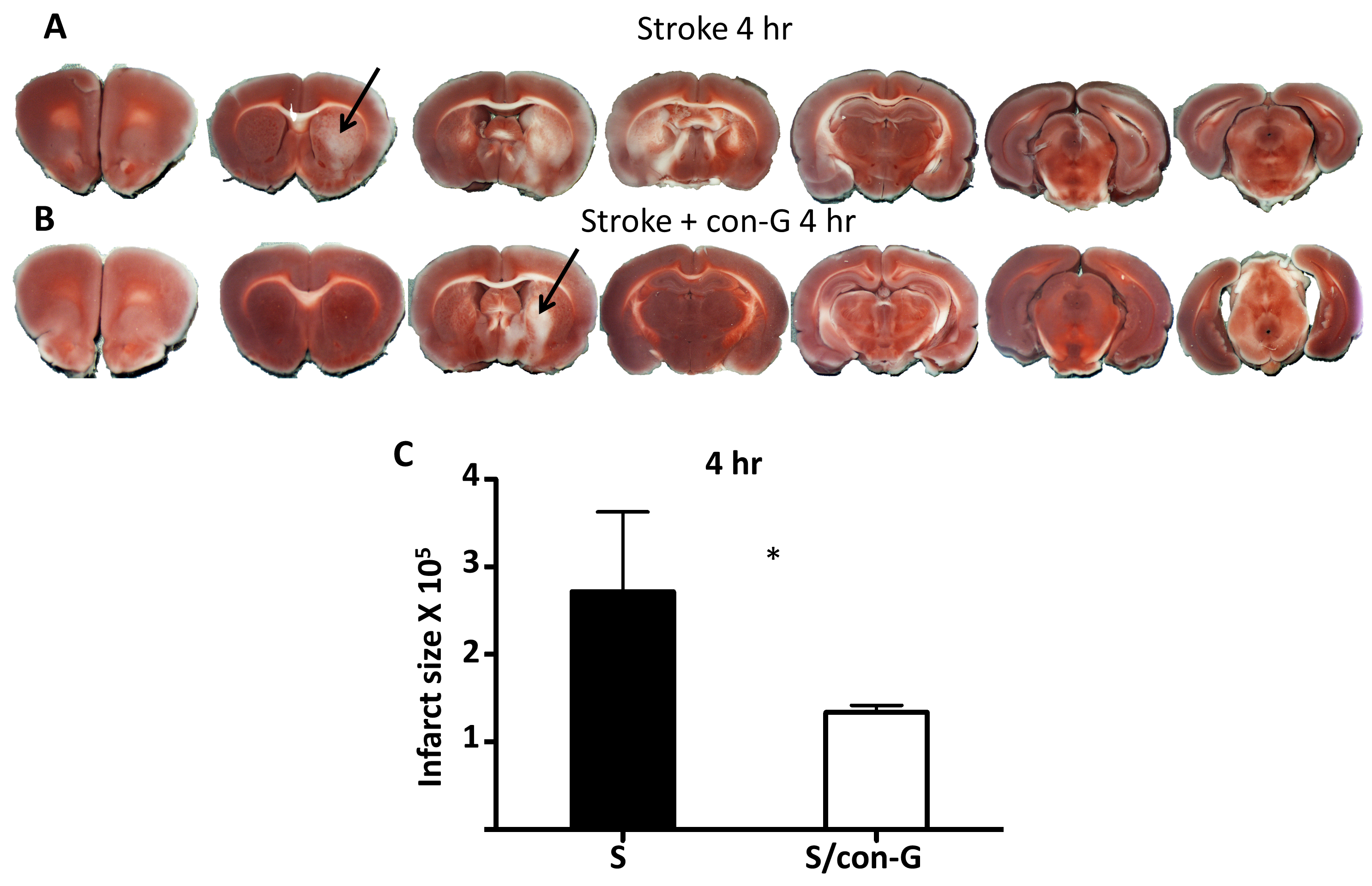

Supplement: S1 Fig — (A) Serial coronal sections of the same brain stained with TTC for non-treated rats and (B) rats treated with 2 μM con-G sacrificed 4 hr post-MCAO. The infarct area is observed in white in the right hemisphere (black arrows). (C) Actual infarct size for non-treated and 2 μM con-G treated rat brains. N = 4 for both S and S/con-G at 4 hr. *p<0.05 between non-treated and con-G-treated groups. (TIF) [file pone.0122840.s001.tif]

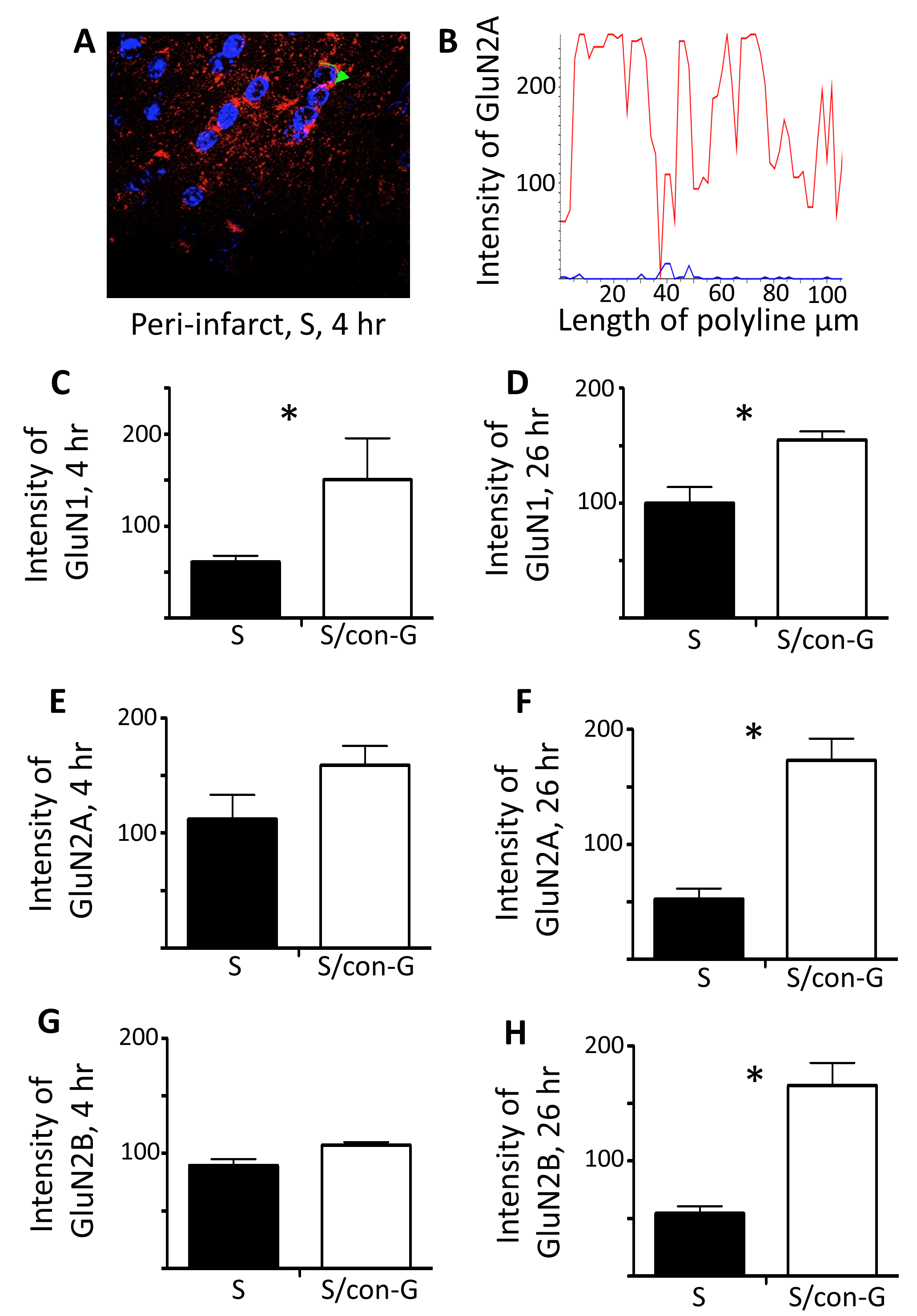

Supplement: S2 Fig — (A) Representative image of GluN2A staining in the peri-infarct region of non-treated rat brains to show the 100 μm polyline for determining fluorescence intensity profile. (B) In the intensity profile, red indicates GluN2A subunit fluorescence intensity over a distance of 100 μm and the blue indicates intensity of the nuclear stain DAPI. Quantification of the GluN1 punctae at 4 hr (C) and 26 hr (D), of GluN2A staining at 4 hr (E) and 26 hr (F), and of GluN2B staining at 4 hr (G) and 26 hr (H). *p<0.05 between non-treated (S) and con-G (S/con-G) treated rat brains. N = 2 for S and n = 7 for S/con-G at 26 hr, and n = 4 for both S and S/con-G at 4 hr. (TIF) [file pone.0122840.s002.tif]
